# Programmed cell death 5 transgenic mice attenuates adjuvant induced arthritis by 2 modifying the T lymphocytes balance

**DOI:** 10.1186/s40659-017-0145-4

**Published:** 2017-12-11

**Authors:** Feng Yuan, Junfeng Wang, Keshi Zhang, Zhao Li, Zhenpeng Guan

**Affiliations:** 1 Arthritis Clinic & Research Center, Peking University People’s Hospital, 11 Xizhimen South Street, Xicheng District, Beijing, 100044, China; 2 Department of Orthopaedics, Peking University International Hospital, Beijing, 102206 China

**Keywords:** Rheumatoid arthritis, Adjuvant-induced arthritis, Programmed cell death 5, T lymphocytes, Inflammatory cytokines

## Abstract

**Background:**

Programmed cell death 5 (PDCD5) is an apoptosis-related gene cloned from TF-1 cells whose primary biological functions are to promote apoptosis and immune regulation. The effects and mechanisms exerted by key mediators of arthritic inflammation remain unclear in PDCD5 transgenic (PDCD5 tg) mice.

**Results:**

In the current study, PDCD5 tg mice inhibited the progression of adjuvant-induced arthritis, specifically decreasing clinical signs and histological damage, compared with arthritis control mice. Additionally, the ratio of CD4^+^IFN-γ^+^ cells (Th1) and CD4^+^IL-17A^+^ cells (Th17), as well as the mRNA expression of the pro-inflammatory mediators IFN-γ, IL-6, IL-17A and TNF-α, were decreased in PDCD5 tg mice, while CD4^+^CD25^+^Foxp3^+^ regulatory T (Treg) cells and the anti-inflammatory mediators IL-4 and IL-10 were increased. Furthermore, PDCD5 tg mice demonstrated reduced serum levels of IFN-γ, IL-6, IL-17A and TNF-α and increased levels of IL-4.

**Conclusions:**

Based on our data, PDCD5 exerts anti-inflammatory effects by modifying the T lymphocytes balance, inhibiting the production of pro-inflammatory mediators and promoting the secretion of anti-inflammatory cytokines, validating PDCD5 protein as a possible treatment for RA.

## Background

Rheumatoid arthritis (RA), one of the most common autoimmune diseases worldwide, is pathologically characterized by chronic inflammation of the synovium followed by pannus formation, ultimately leading to cartilage and bone erosion [1]. Although the aetiology and pathogenesis of RA have not been confirmed, immune factors, particularly T lymphocyte-mediated autoimmune responses, are involved in disease progression [2]. T lymphocyte participation in the initiation and progression of RA results in the release of a variety of pro-inflammatory mediators and the inhibition of anti-inflammatory cytokines, contributing to increased inflammation and destruction of the affected joint [3, 4]. Thus, restoration of immune homeostasis via modulation of the balance between T lymphocytes, which can control or even eliminate inflammatory responses, is an optimal strategy for RA therapy.

PDCD5 is an apoptosis-related gene cloned from TF-1 cells that is evolutionarily conserved and widely expressed in vivo [5]. The molecular mechanism by which PDCD5 accelerates cell apoptosis primarily involves the Tip60-p53 signalling pathway [6]. Restoration of normal levels of PDCD5 in vivo can enhance the sensitivity of various tumour cells to chemotherapeutic drugs by inducing apoptosis [7–9]. Furthermore, growing numbers of researchers have focused on another important biological function of PDCD5: immune regulation. The abnormal expression of PDCD5 has been detected in many autoimmune diseases, such as RA, psoriasis and hepatitis [10–12]. For example, in our preliminary studies, PDCD5 levels in plasma and synovial fluid were inversely associated with pro-inflammatory cytokines (TNF-α and IL-17) and disease activity in patients with RA [13, 14].

Adjuvant-induced arthritis (AIA), a well-established animal model of RA, has been extensively used to identify potential anti-arthritic agents in transgenic and knockout animals [15]. A single subcutaneous injection of adjuvant in the plantar hindpaw induces rheumatoid-like joint inflammation and destruction with a peak phase 14 days after immunization. Recombinant PDCD5 protein intraperitoneal injection can protect the joint against inflammatory destruction evoked by collagen-induced arthritis in rats [16]. However, the effects and mechanisms exerted by key mediators of arthritic inflammation in PDCD5 tg mice, in which the PDCD5 protein is highly expressed in CD4^+^ T cells, remain unclear [17].

In the present study, we have demonstrated the crucial role of PDCD5 in attenuating the development of AIA. Decreased arthritis score, joint circumference, paw oedema and histological damage were observed in PDCD5 tg mice compared with arthritic control mice. Additionally, PDCD5 tg mice increased Treg proportion and decreased Th1/Th17 ratio. Furthermore PDCD5 tg mice exerted anti-inflammatory effects by inhibiting the production of pro-inflammatory mediators (IFN-γ, IL-6, IL-17A and TNF-α) and promoting the secretion of anti-inflammatory cytokines (IL-4 and IL-10).

## Methods

### Induction and evaluation of AIA

Male C57BL/6J mice (8–10 weeks old) were purchased from Vital River Laboratory (Beijing, China). Age-matched PDCD5 tg mice with the C57BL/6J background were kindly donated by Peking University Center for Human Disease Genomics [17]. The presence of the human PDCD5 gene was confirmed by performing PCR with tail DNA. Both wild-type and transgenic mice were bred under specific pathogen-free conditions at the Experimental Animal Center, Peking University People’s Hospital (Beijing, China). The mice were anaesthetized with isoflurane and euthanized via cervical dislocation. All experimental procedures used in our study were approved by the Institutional Animal Care and Use Committee of Peking University People’s Hospital. The experimental animal were divided into four groups, each group contains 6 mice. The normal control group (NC) and the arthritis control group (AC) were used wild-type mice; while the transgenic control group (TC) and the transgenic arthritis group (TA) were used PDCD5 tg mice. CFA was purchased from Chondrex (USA) and AIA was induced as previously described [15]. AC and TA were immunized subcutaneously in the left plantar hindpaw with a 20 μl injection of CFA containing 5 mg/ml heat-killed mycobacteria. NC and TC were injected in the same location with 20 μl of phosphate-buffered saline. Measurements of joint circumference and paw oedema were performed using a tape measure and digital callipers, respectively. The arthritis score was periodically observed to evaluate the severity of arthritis after the injection of CFA for 14 days by two independent researchers. The progression of arthritis was assessed using the following criteria, ranging from 0 to 4: 0, no erythaema or swelling; 1, joint erythaema and no swelling; 2, joint erythaema and mild swelling; 3, joint erythaema and moderate swelling; 4, severe swelling with dysfunction [18].

### Histology

After euthanasia on day 14 following CFA injection, the tibiotarsal joints were removed. The tissue samples were fixed in 4% formalin for 48 h, decalcified in 10% EDTA solution for 4 weeks, embedded in paraffin and sectioned (5 μm). Haematoxylin and eosin (H&E) staining was applied to investigate the degree of joint inflammation and destruction. The histological scores of the tibiotarsal joints were assessed by two blinded and independent pathologists using the following criteria. The score were defined on a scale of 0–4 point: 0, no inflammation; 1, minimal inflammatory infiltration; 2, mild inflammatory infiltration and synovial hyperplasia; 3, moderate inflammatory infiltration and pannus formation; 4, severe inflammatory infiltration, cartilage and bone erosion [16].

### Flow cytometry analysis

Spleens were harvested on the day of euthanasia to prepare single cell suspensions via passage through mesh screens. The splenocytes were washed with red blood cell lysis buffer (BioLegend, USA) and then washed twice with PBS. To perform intracellular labelling, cells (2 × 10^6^ per sample) were incubated with Cell Stimulation Cocktail (eBioscience, USA) for 5 h at 37 °C. Subsequently, the cells were surface-stained with a FITC-labelled CD4 monoclonal antibody (eBioscience), then fixed and permeabilized using an Intracellular Fixation and Permeabilization Buffer Set (eBioscience), followed by intracellular staining with a PerCPCy5.5-labelled IFN-γ monoclonal antibody (eBioscience) and an APC-labelled IL-17A monoclonal antibody (eBioscience). To determine Treg frequency, the cells (2 × 10^6^ per sample) were surface-stained with a FITC-labelled CD4 monoclonal antibody (eBioscience) and an APC-labelled CD25 monoclonal antibody (eBioscience). After fixation with the Intracellular Fixation and Permeabilization Buffer Set (eBioscience), the cells were stained with a PE-labelled FoxP3 monoclonal antibody (eBioscience). Isotype-matched IgG antibody (eBioscience) was used as a negative control. The stained cells were analysed using a FACSCalibur flow cytometer (BD Biosciences, USA) with FlowJo software version 7.6.

### RNA extraction and real-time PCR

All extraction procedures were performed on ice using ice-cold reagents. Total RNA was isolated from spleens using a total RNA extraction kit (Tiangen Biotech, Beijing, China) and all procedures comply with the manufacturer’s instructions. The obtained mRNA was quantified by measuring the absorbance at 260 nm, and its quality was determined by measuring the 260/280 ratio. Complementary DNA synthesis was performed using a Fast Quant RT kit (Tiangen Biotech) in accordance with the manufacturer’s instructions. The mRNA expression levels of target genes in the spleen were determined by performing real-time PCR using SuperReal PreMix Plus (Tiangen Biotech) on a Bio-Radi Cycler Opnion Monitor3 System (USA). GAPDH was used as an internal control, and each reaction was performed in triplicate. The real-time PCR data were analysed using the 2^−ΔΔCt^ method. The primers used in these assays were selected from the PubMed database. Primer sequences (5′–3′) that were used are: IFN-γ (5′-TCTGGGCTTCTCCTCCTGCGG-3′,5′-GGCGCTGGACC TGTGGGTTG-3′), IL-4 (5′-GAAGCCCTACAGACGAGCTCA-3′,5′-ACAGGAGAAGGGA CGCCAT-3′), IL-6 (5′-CCGGAGAGGAGACTTCACAG-3′,5′-GGAAATTGGGGTAGGAAGGA-3′), IL-10 (5′-ACCTGCTCCACTGCCTTGCT-3′,5′-GGTTGCCAAGCCTTATCGGA-3′), IL-17A (5′-ATCCCTCAAAGCTCAGCGTGTC-3′,5′-GGGTCTTCATTGCGGTGGAGAG-3′), TNF-α (5′-GCGGAGTCCGGGCAGGTCTA-3′,5′-GGGGGCTGGCTCTGTGAGGA-3′), GAPDH (5′-CCCAGCAAGGACACTGAGCAAG-3′,5′-GGTCTGGGATGGAAATTGTGAGGG-3′).

### Cytometric bead array (CBA)

Serum samples were obtained by collecting retro-orbital plexus blood. Cytokine levels were measured using a mouse Th1/Th2/Th17 CBA kit (BD Biosciences, USA) according to the manufacturer’s instructions. Concentrations were calculated based on mean fluorescence intensity values, which were detected using a BD FACS Aria II flow cytometer (BD Biosciences, USA). The data were analysed using BD Cytometric Bead Array analysis software.

### Statistical analysis

All data are expressed as the mean ± SEM. Differences between the experimental groups were tested using Student’s t test or Two-way Anova by SPSS 20.0 (USA). And *p* < 0.05 were considered statistically significant.

## Results

### PDCD5 tg mice attenuates the development of AIA and protects the joint against inflammatory destruction

**Fig. 1:**
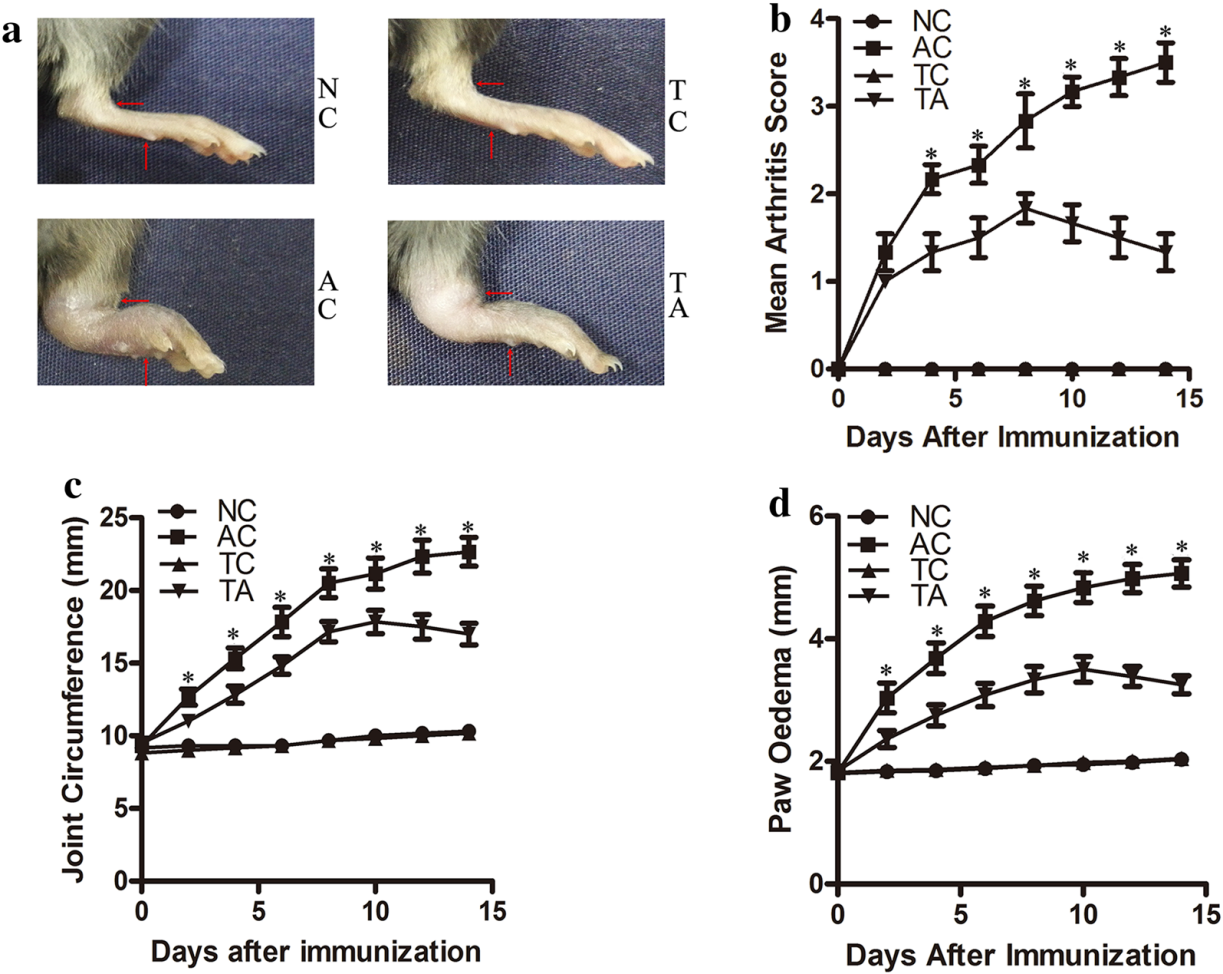
PDCD5 tg mice attenuates the development of AIA. **a** Photographs showing the left plantar hindpaws of mice from each experimental group. The red arrows indicate the measurement position for joint circumference and paw oedema, respectively. **b** The arthritis score during the development of AIA. **c** Changes in joint circumference after CFA immunization. **d** Paw oedema after CFA immunization. Due to NC and TC as normal control, the value of arthritis score, joint circumference and paw oedema are very close, the numerical curves of the two groups are overlap in **b**–**d** (*p < 0.05, TA vs AC)

**Fig. 2:**
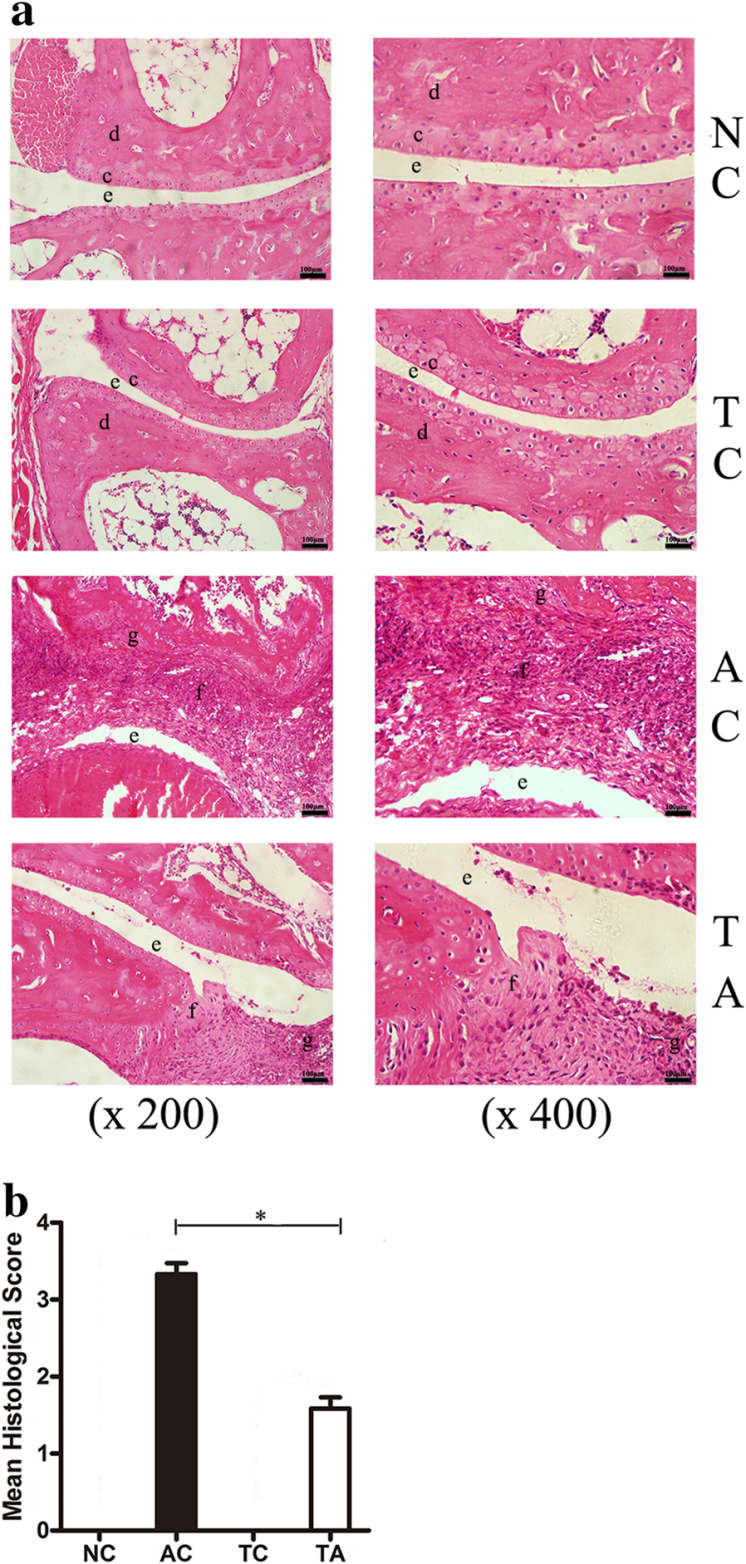
Protective effects of PDCD5 tg mice on the histological changes in AIA. **a** Representative sections of joint histopathology are shown. Histological examination of tibiotarsal joint sections stained with H&E under a light microscope at ×200 and ×400 magnification, respectively. **b** Histological scores of tibiotarsal joints. (c) Cartilage. (d) Bone. (e) Joint gap. (f) Inflammatory infiltration. (g) Synovial hyperplasia. (*p < 0.05, TA vs AC)

To validate the anti-inflammatory effects of PDCD5 on AIA mice, clinical signs and pathological changes were first observed to evaluate arthritis severity. PDCD5 tg mice did not demonstrate a reduced incidence of AIA; however, significant reductions in arthritis score, joint circumference and paw oedema were observed compared with arthritis control mice (Fig. 1). Additionally, the average histological score was lower in PDCD5 tg mice due to significant reductions in inflammatory cell infiltration, synovial hyperplasia, and cartilage and bone erosion, consistent with the observed clinical symptoms (Fig. 2). Based on these data, the transfer of PDCD5 gene can relieve inflammatory joint destruction in murine AIA.

### PDCD5 tg mice increases Treg proportion and decreases the Th1 and Th17 ratio

**Fig. 3:**
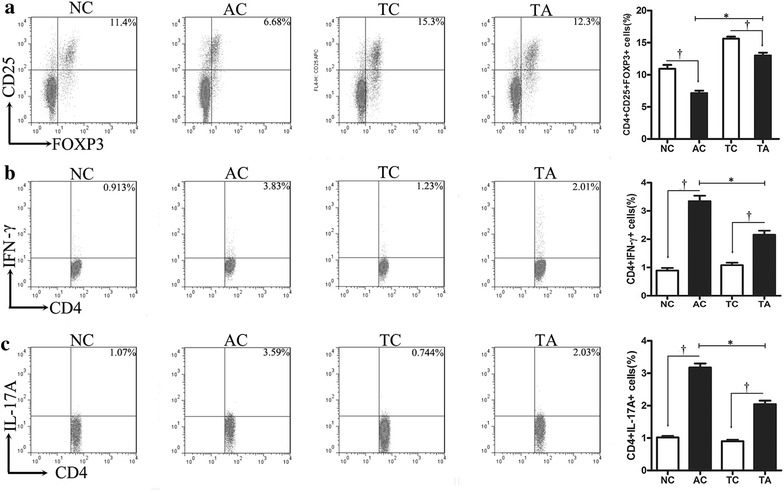
PDCD5 tg mice increases Treg proportion and decreases the Th1 and Th17 ratio. **a** Representative plots and quantification of CD4^+^CD25^+^Foxp3^+^ (Treg) cells in the spleen were analysed by flow cytometry. **b** Representative plots and quantification of CD4^+^IFN-γ^+^(Th1) cells in the spleen analysed by flow cytometry. **c** Representative plots and quantification of CD4^+^IL-17A^+^ (Th17) cells in the spleen analysed by flow cytometry (^†^ *p* < 0.05, AC vs NC and TA vs TC respectively; *p < 0.05, TA vs AC)

Because the relationships between T lymphocytes are disturbed during the pathological processes in AIA, we studied the percentages of Treg, Th1 and Th17 among splenocytes using flow cytometry. The ratio of Treg in AC and TA group were decreased compared with their normal control after CFA immunized, however Treg levels in PDCD5 tg mice was significantly higher than AC group. Next, we found that the proportion of Th1 and Th17 in PDCD5 tg mice and their arthritis control were increased compared than TC and NC, respectively. But, the elevated proportion in PDCD5 tg mice was significantly lower than AC group (Fig. 3). Thus, PDCD5 tg mice attenuate AIA by increasing the proportion of Treg and decreasing the ratio of Th1 and Th17.

### PDCD5 tg mice downregulates the mRNA expression of pro-inflammatory mediators and upregulates anti-inflammatory mediators

**Fig. 4:**
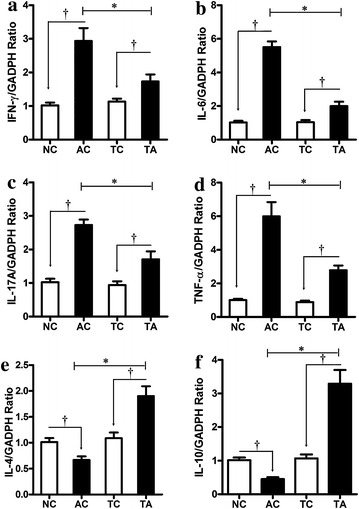
PDCD5 tg mice regulates the gene expression of pro-inflammatory and anti-inflammatory mediators in the spleen. Relative mRNA expression of IFN-γ (**a**), IL-6 (**b**), IL-17A (**c**), TNF-α (**d**), IL-4 (**e**) and IL-10 (**f**) was determined by Real Time PCR in AIA spleens (^†^ *p* < 0.05, AC vs NC and TA vs TC respectively; *p < 0.05, TA vs AC)

As the largest secondary lymphoid organ, the spleen contains a large number of reserve T lymphocytes. Therefore, we next investigated the effects of PDCD5 on the expression of inflammatory mediators. Compared with their normal control, mRNA expression of the pro-inflammatory mediators IFN-γ, IL-6, IL-17A and TNF-α was increased in AC and TA group, however PDCD5 tg mice can relatively reduce the expression of these pro-inflammatory mediators, thereby inhibiting the inflammatory response in vivo. Furthermore, decreased levels of IL-4 and IL-10 were observed in AC group compared with NC group. But, the expression of these anti-inflammatory mediators in PDCD5 tg mice were significantly higher than NC and TC group (Fig. 4).Therefore, PDCD5 tg mice play a protective role in AIA may not only inhibit the expression of pro-inflammatory mediators, but also promote the expression of anti-inflammatory mediators.

### Effects of PDCD5 tg mice on serum cytokine production

**Fig. 5:**
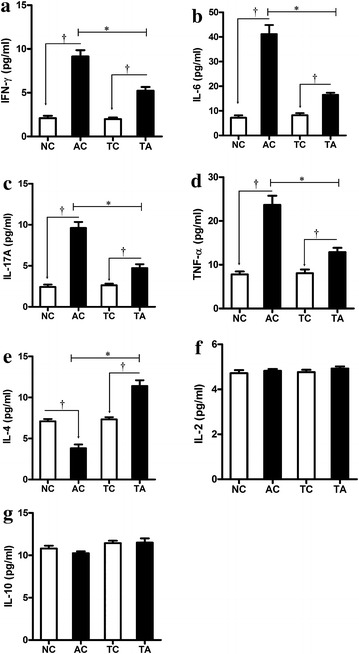
Effect of PDCD5 tg mice on inflammatory cytokines in serum. **a** IFN-γ levels; **b** IL-6 levels; **c** IL-17A levels; **d** TNF-α levels; **e** IL-4 levels; **f** IL-2 levels; **g** IL-10 levels (^†^ *p* < 0.05, AC vs NC and TA vs TC respectively; *p < 0.05, TA vs AC)

Because cytokines directly influence the immune responses exerted by T lymphocytes, we examined the levels of pro-inflammatory and anti-inflammatory cytokines in AIA mice. Since the immune function of CFA, the serum levels of IFN-γ, IL-6, IL-17A and TNF-α in PDCD5 tg mice and their arthritis control were significantly increased, however the production of pro-inflammatory cytokines in PDCD5 tg mice were relatively inhibited. Simultaneously, the expression of IL-4 in PDCD5 tg mice was higher than their normal control and arthritis control. However, there were no differences in the levels of serum IL-2 and IL-10 among these groups in our experiment (Fig. 5). Therefore, PDCD5 exerts immunosuppressive effects partly by regulating the balance between pro-inflammatory and anti-inflammatory cytokines.

## Discussion

In the present study, PDCD5 tg mice demonstrated protective effects on clinical symptoms during AIA compared with arthritis control mice. The anti-inflammatory effects of PDCD5 tg mice were further validated by histological examination. Furthermore, the ratio of Th1 and Th17, as well as the mRNA expression of the pro-inflammatory mediators IFN-γ, IL-6, IL-17A and TNF-α, were decreased in PDCD5 tg mice. Increased levels of Treg and the anti-inflammatory mediator IL-4 and IL-10 were observed in PDCD5 tg mice. In addition, compared with arthritis control, PDCD5 tg mice reduced serum levels of the pro-inflammatory cytokines IFN-γ, IL-6, IL-17A and TNF-α and upregulated the expression of the anti-inflammatory cytokine IL-4. These findings provide theoretical support for anti-inflammatory effects of PDCD5.

Accumulating evidence indicates that T lymphocytes mediated immune responses play a critical role in the pathogenesis and progression of RA [2]. T lymphocytes represent a major research tool to develop new anti-inflammatory agents to control the symptoms of RA in a more effective and safe manner [19]. Previous studies supported the contribution of the dominant immune activation of Th1 over Th2 cells, accompanied by the dysregulated production of inflammatory mediators, to the pathogenesis of RA [20, 21]. Th1 and Th2 cells, two classical CD4^+^ T cells, regulate the cellular immune response and the humoral immune response, respectively. Pro-inflammatory cytokines such as IFN-γ, IL-6 and TNF-α, which contribute to inflammatory reactions and immunological destruction, are primarily released by Th1 cells. In contrast, Th2 cells produce the anti-inflammatory cytokines IL-4 and IL-10. Increased expression of IL-4 and IL-10 drives the balance towards the Th2 immune response by suppressing inflammation and proliferation of the synovium, inhibiting the secretion of pro-inflammatory cytokines, and promoting the production of various antibodies [21]. In our study, PDCD5 inhibited the production of IFN-γ, IL-6 and TNF-α and promoted the secretion of IL-4 and IL-10, indicating a shift towards a Th2 dominant response. This partially explains the protective effects of PDCD5 on adjuvant-induced arthritis in mice.

With the deepening of RA research, many studies have found Th17 cells, another type of helper T cells, are essential for the persistent and progressive joint inflammation [22, 23]. The pro-inflammatory cytokine IL-17, which contributes to synovial inflammation and bone erosion, is predominantly secreted by Th17 cells [23]. The elevated expression of IL-17 in the synovium and serum is proportional to disease activity in RA patients [24]. Additionally, increased levels of IL-17 were also observed in experiments with RA animals [23, 25]. The ability to block pro-inflammatory cytokines, such as TNF-α and IL-17, would elevate the efficacy of new treatment agents evaluated in RA animal experiments and clinical trials [26, 27]. Treg cells, which are essential for the regulation of autoimmunity and inflammation, are identified by their expression of the transcription factor Foxp3 [28]. Treg cells secrete anti-inflammatory cytokines such as IL-10 and TGF-β to promote auto-reactive T cell apoptosis and establish immune tolerance. Treg dysfunction can influence the balance of various T lymphocytes and inflammatory factors, contributing to inflammatory destruction in AIA [29]. Additionally, some potential anti-arthritic agents, which can exhibit various protective effects, like reducing neutrophil migration, mechanical hypernociception, and proteoglycan loss, are associated with increased Treg proportion [30]. The use of Treg cells as a therapeutic method prolongs the remission period of patients with RA [31, 32]. The increased Th17/Treg ratio can promote the expression of IL-17 in the development of AIA, eventually leading to persistent inflammation and bone destruction [25]. Thus, restoration of the balance between Th17 and Treg cells, a crucial indicator of immune homeostasis, represents a promising target for RA treatment. In our study, PDCD5 tg mice exhibited decreased numbers of Th17 cells and increased Treg cells, thus inhibiting the secretion of IL-17 and promoting the secretion of IL-10. Besides, attenuated clinical symptoms and pathological changes were observed in AIA mice. The Th17/Treg ratio was markedly decreased in PDCD5 tg mice compared with that in arthritic control mice, providing an additional explanation for the mechanism underlying the anti-inflammatory properties of PDCD5.

## Conclusion

In summary, PDCD5 attenuates joint inflammation and destruction in AIA mice. The anti-inflammatory properties of PDCD5 appear to be mediated through increasing the Th1 to Th2 immune response, decreasing the Th17/Treg ratio and regulating the inflammatory cytokine imbalance. Therefore, PDCD5 protein may be a promising therapeutic agent for the treatment of RA.

## Abbreviations

AIA: adjuvant-induced arthritis
CFA: complete Freund’s adjuvant
EAE: encephalomyelitis
GAPDH: glyceraldehyde 3-phosphate dehydrogenase
HE: haematoxylin and eosin
IFN: interferon
IL-: interleukin-
MS: multiple sclerosis
PBS: phosphate-buffered saline
PDCD5: programmed cell death 5
RA: rheumatoid arthritis
Rh: recombinant human
Tg: transgenic
TGF: transforming growth factor
Th: T helper cells
TNF: tumor necrosis factor
Treg: regulatory T cells
